# Antimicrobial activity of amazonian medicinal plants

**DOI:** 10.1186/2193-1801-2-371

**Published:** 2013-08-05

**Authors:** Amanda A Oliveira, Jorge FO Segovia, Vespasiano YK Sousa, Elida CG Mata, Magda CA Gonçalves, Roberto M Bezerra, Paulo OM Junior, Luís IB Kanzaki

**Affiliations:** Laboratory of Bioprospection, University of Brasília, University Campus Darcy Ribeiro, Asa Norte, Brasília, DF Brazil; Brazilian Agricultural Research Corporation/Amapá, Rod. J K, 05 km, number 2600, University District, Macapá, AP Brazil; Amapa State University, Presidente Vargas Avenue, number 650, Center, Macapá, AP Brazil; Federal University of Amapá, Rod Juscelino Kubitschek, KM-02, Jardim Marco Zero, Macapá, AP Brazil

**Keywords:** Antimicrobials, Environment, Medicinal plants, Metabolites, Amazon

## Abstract

**Objectives:**

The aqueous extracts of currently utilized Amazonian medicinal plants were assayed *in vitro* searching for antimicrobial activity against human and animal pathogenic microorganisms.

**Methods:**

Medium resuspended lyophilized aqueous extracts of different organs of Amazonian medicinal plants were assayed by *in vitro* screening for antimicrobial activity. ATCC and standardized microorganisms obtained from Oswaldo Cruz Foundation/Brazil were individually and homogeneously grown in agar plate, and holes previously perforated in the gel were filled with diluted plant aqueous extracts. Inhibition halos were evaluated and controlled by the use of the fluoroquinolone ciprofloxacin.

**Results:**

The Amazonian medicinal plants, *Hymenelobium petraeum* showed inhibitory activity over *Staphylococcus aureus, Enterococcus faecalis*, *Salmonella enterica* serovar Typhi*, Acinetobacter baumannii* and *Candida albicans,* while *Vatairea guianensis* and *Symphonia globulifera* presented inhibitory activity exclusively for *Staphylococcus aureus*. Also, *Ptychopetalum olacoides* and *Pentaclethra macroloba* inhibited the growth of *Klebsiella ozaenae* and *Acinetobacter baumannii.*

**Conclusion:**

The aqueous botanic extracts that showed activity against microroganisms of ATTC and Osvaldo Cruz strains had at least 40% of antimicrobial activity when compared to halo inhibition produced by the commercial antibiotic ciprofloxacin utilized as a control. Of all plants extracts assayed, the *Hymenelobium petraeum* had the best performance, sometimes exhibiting higher activity than ciprofloxacin. It is not well-defined by the physicians the exact indication of the majority of medicinal plants in the Amazon area in Brazil. Natives utilize the plants according to their symptoms, based on the traditional knowledge transmitted orally from generation to generation, among Amerindians, Afrodescendents and ethnic mixed populations. A significant number of Amazonian medicinal plants are totally unknown related to their medicinal properties including mechanism of action and therapeutic effects, as very few information is reported in the scientific literature. A tiny amount of data is presented, as the preliminary antimicrobial properties of the medicinal plants here accessed, under the urgent necessity of new antibiotics in the market and in face of the increased resistance of infectious microorganisms to antimicrobials.

## Introduction

Nowadays, an increasing number of infectious agents are becoming more resistant to commercial antimicrobial compounds (Hancock et al. 2012). The necessity to develop new drugs requires varied strategies, among them, the bioprospection of secondary metabolites produced by medicinal plants (Dionisi et al. 2012; Benko-Iseppon and Crovella 2010).

It is a common sense that the extensive Amazonian biodiversity, scarcely explored the economic rationality, would yield uncouns opportunities to find plant species potentially secreting metabolites, exhibiting antimicrobial activity, among other medicinal properties (Vieira et al. 2008; Basso et al. 2005).

Despite the Amazonian biome be characterized by high humidity and temperature during all the year, encompassing the rain forest, the limits in the northern region, in the Amapa state, exhibit a transitional area consisting of the Guyana plateau, which enrich much more the biological and geographycal diversity of this area (Alves 2008; Brothwell 1967).

Previously, we corroborated the biological activity of many different medicinal plants collected in distinct geographical regions, in the Amapa state. The ethanolic extracts of *Geissospermum argenteum*, *Uncaria guianensis*, *Brosimum acutifolium*, *Copaifera reticulate*, *Licania macrophylla*, *Ptycopetalum olacoides* and *Dalbergia subcymosa* exhibited inhibitory activity against multiresistant and *Staphylococcus aureus* and *Pseudomonas aeruginosa* ATCC strains (Correia et al. 2008). Also, ethanolic extracts of *Copaifera reticulata, Tabebuia serratifolia, Brosimum rubescens* and *Carapa guianensis* inhibited the colony formation of gram positive bacilli of Bacillus gender and *Pseudomonas aeruginosa*, microorganisms isolated from biocorroded metallic structures in a hydroelectric power unit, in the Amapa state (Correia et al. 2010). In other work, we detected allelopathic activity of ethanolic botanical extracts of *Stryphnodendron adstringens, Carapa guianensis* and *Ouratea hexasperma* utilizing *Lactuca sativa* as the model organism in the assays (Bezerra et al. 2010). Phytochemical analysis of *Manilkara huberi* (Ducke) fam. *Sapotaceae*, autochthonous of Amapa state, detected the presence of alfa and beta-amiriyn compounds (Segovia et al. 2011). Among the species of the Fabaceae, Caesalpiniaceae, Olacaceae, Chrysobalanaceae, Apocynaceae, Rubiaceae and Clusiaceae families, collected in the Amapa state, we detected antiretroviral, and lymphoproliferative activity, and also cytotoxicity for lymphoma/leukemia derived cell lines (Mata 2011).

Here, we investigated the antimicrobial activity of medicinal plants, currently used by native amazonians to treat their ailments. Plants were collected in different areas of Amapa state, in the Amazon region of Brazil.

## Material and methods

### Botanical aqueous extracts

A total of 19 plants aqueous extracts of a collection from the Laboratory of Bioprospection, of medicinal plants collected in the Amapa state, encompassing the Fabaceae (members of Caesalpiniaceae subfamily), Rubiaceae, Ochnaceae, Apocynaceae and Clusiaceae families were utilized searching for biological activity. The provisional voucher number of the plants here studied are kept at the Brazilian Agricultural Research Corporation/EMBRAPA, until be definitely transferred to the herbarium of the Institute of Scientific and Technological Research of the Amapa State (IEPA), Brazil. The aqueous extracts were prepared as described in Mata (2011). Briefly, 100 grams of powdered botanical sample (leaves, bark or fruits) were added to 1 L of distilled water at 70°C and allowed to cool until reaching 40°C in a water bath. Sequentially, the solution was filtered in filter paper, frozen at −20°C and lyophilized in a Liotop apparatus (Model l202, Liobras, Brazil). Before use, lyophilized samples were diluted in RPMI 1640 incomplete medium (INLAB, Brazil) and utilized in concentration of 2.275 mg/mL.

### ATCC and Oswaldo Cruz Foundation Pathogenic Microorganisms

The antimicrobial activity was assayed utilizing two groups of well-known microorganisms. One group of ATCC gram negative, pathogenic strains: *Escherichia coli* 25922*, Escherichia coli* 35218*, Salmonella enterica serovar* Enteritidis 564*, Salmonella enterica* serovar Typhimurium 5190*, Pseudomonas aeruginosa* 27853 and ATCC gram positive, pathogenic strains: *Staphylococcus aureus* 25923 and *Enterococcus faecalis* 29212; another group, gram negative microorganisms from Oswaldo Cruz Institute Foundation: *Escherichia coli* 00219; *Salmonella enterica* serovar Typhi *0029; Acinetobacter baumannii* 00143; *Klebsiella ozaenae* 0075, the gram positive *Enterococcus faecalis* 00531 and, the yeast *Candida albicans* 40006. The microorganisms were maintained in nutrient agar at 4°C until the assays were carried out. All samples were kindly donated by Dr. Ernesto Hofer from Oswaldo Cruz Foundation (FIOCRUZ), Rio de Janeiro, Brazil.

### Agar diffusion test

In order to assess the inhibitory activity of the botanical aqueous extracts, 10 uL of each cultured microorganism in 1X10^-3^ UFC/mL was plated in Muller-Hinton agar, which was previously perforated yielding 1 central hole surrounded by 8 ones. In the central hole, 30 uL of ciprofloxacin (16.6 ug/mL) were added, and in the surrounded holes, 30 uL of each plant aqueous extract. Antimicrobial activity was assessed by the appearance of inhibitory halo around the hole, without microbial growth.

## Results

**Figure 1:**
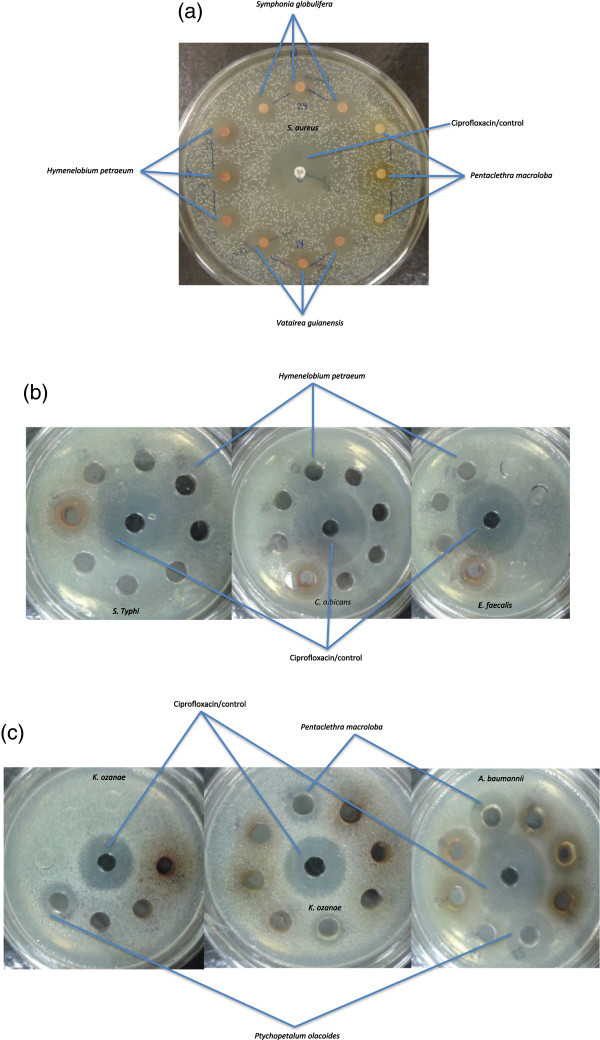
**Antimicrobial activity of amazonian plants. (a)***S. aureus* inhibited by extracts of *H. petraeum*, *S. globulifera* and *Vatairea guianensis***(b)** Inhibition of *S. enterica* serovar Typhi, *C. albicans* and *E. faecalis* by extract of *H. petraeum***(c)** Extracts of *P. olacoides* and *P. macroloba* inhibited *Klebsiella ozaenae* and *Acinetobacter baumannii*.

The botanical aqueous extract of *Hymenelobium petraeum* (provisional voucher number BRM54) inhibited the colony formation of *Staphylococcus aureus* ATCC 25923, *Enterococcus faecalis* ATCC 29212, *Salmonella enterica* serovar Typhi *0029* and *Candida albicans*, while the aqueous extracts of *Vatairea guianensis* (provisional voucher number BRM27) and *Symphonia globulifera* (provisional voucher number ESK71) were active against *Staphylococcus aureus*. Also, the aqueous extract of *Ptychopetalum olacoides* (provisional voucher number BRM08) and *Pentaclethra macroloba* (provisional voucher number BRM36) inhibited the growth of *Klebsiella ozaenae* and *Acinetobacter baumannii.* In all cases (Figure 1a, b and c), the botanical extract concentration utilized was 2.275 ug/uL and the initial inoculum of microorganisms was 1X10^-3^ UFC/mL of ATCC bacterial strains and FIOCRUZ strains.

## Discussion

Similar results to what is described in this study were found by (Araújo 2010), assaying ethanolic extracts of *V. guianensis* and *S. globulifera*, describing inhibitory activity over *S. aureus* colony’s growth, obtained from human patients with oral mucositis. Furthermore, *S. globulifera* extract exhibited activity against the growth of all known species of Streptococcus gender and *Candida albicans*. In Dr. Duke’s Phytochemical and Ethnobotanical Databases, *V. guianensis* is indicated for the treatment of ringworm and sore (Dr. Duke’s Phytochemical and Ethnobotanical Databases. 2012. http://www.ars-grin.gov/cgi-bin/duke/ethnobot.pl?Vatairea%20guianensis), and *S. globulifera* for the treatment of headache and ulcer. There are not any data concerning the phytochemical analysis of *Hymenelobium petraeum* in the literature, except studies describing the plant anatomy and physiology (Oliveira et al. 2010). Here, we present the first report concerning the antimicrobial action of *Ptychopetalum olacoides* and *Pentaclethra macroloba*. The aphrodisiac properties of *Ptychopetalum olacoides* are well-known among native people in the Amazon forest. Based on these folk beliefs, some studies were carried out to investigate the biological activity of this plant in the nervous system, for the treatment of stress, sexual dysfunction and possibly Alzheimer disease (Mendes and Carlini 2007; Howes and Houghton 2012). Few studies describe the antihemorrhagic and antiproteolytic activity of *Pentaclethra macroloba* (da Silva et al. 2007).

Previously, we reported the antimicrobial activity of *Dalbergia subcymosa*, and now we confirm the biological property with other microorganisms (Correia et al. 2008). There is not any other published data about *Dalbergia subcymosa.*

## Conclusions

Considering that 9 mm halo inhibition produced by the commercial antibiotic ciproflacin (test control) over *Staphylococcus aureus* represents 100% of antibacterial activity, the botanic aqueous extracts of *Vatairea guianensis* had 44.4% (4 mm) activity while *Symphonia globulifera* and *Hymenelobium petraeum* (3.66 mm) 40.6%. In the same way, the botanic aqueous extract of *Pentaclethra macrolaba* did not show any activity over *Staphylococcus aureus*, but inhibited the growth of *Klebsiella ozaenae* and *Acinetobacter baumannii*, representing 49.5% (3.96 mm halo) and 52.4% (5.76 mm halo) respectively, of the ciprofloxacin halo produced (8 mm and 11 mm halo respectively). Also, the botanic aqueous extract of *Ptychopetalum olacoides* inhibited exclusively the growth of *Klebsiella ozaenae* and *Acinetobacter baumannii*, producing halos higher than 100% (9.25 mm halo) and 45.4% (5 mm halo) respectively, when compared to halos produced by ciprofloxacin (8 and 11 mm). Of all aqueous botanical extract tested, *Hymenelobium petraeum* was the most active, exhibiting also activity against *Salmonella enterica* ser. Typhi (7 mm halo), *Candida albicans* (11.9 mm halo) and *Enterococcus faecalis* (5 mm halo) representing 70%, 87.3% and 45.5% respectively, when compared to ciprofloxacin activity (10 mm, 13.63 mm and 11 mm halo respectively) (Table 1).

**Table 1:** **Halo measurement of antimicrobial activity of amazonian plant aqueous extracts**

Botanical extract	Gram positive bacteria	Median and Standard deviation (halo size)
*Symphonia globulifera*	*Staphylococcus aureus*	3.66 mm ± 0.76
*Vatairea guianensis*		4.0 mm ± 0.5
*Hymenelobium petraeum*		3.66 mm ± 0.57
Ciprofloxacin		9 mm ± 0
	**Gram negative bacteria**	
*Hymenelobium petraeum*	*Salmonella enterica* ser. Typhi	7 mm ± 0.26
Ciprofloxacin		10 mm ± 0
*Hymenelobium petraeum*	*Enterococcus faecalis*	5 mm ± 0.1
Ciprofloxacin		11 mm ± 0
*Pentaclethra macrolaba*	*Klebsiella ozanae*	3.96 mm ± 0.15
*Ptychopetalum olacoides*		9,25 mm ± 0.35
Ciprofloxacin		8 mm ± 1.4
*Pentaclethra macrolaba*	*Acinetobacter baumannii*	5.76 mm ± 0.25
*Ptychopetalum olacoides*		4.9 mm ± 0.14
Ciprofloxacin		11 mm ± 0
	**Yeast**	
*Hymenelobium petraeum*	*Candida albicans*	11.9 mm ± 0.1
Ciprofloxacin		13.63 mm ± 0.32

The preliminary results obtained in these experiments pave the road to explore the potential development of new compounds to be launched in the pharmaceutical market filling a tremendous gap, as day by day new multiresistant microorganisms emerges. All plants here assayed present medicinal properties being commonly utilized by local people in the Amazon region. Therefore it is much easier to confirm the new curative properties found, as the proper use of these plants is for general purposes. Likewise, the dried pieces of *Hymenelobium petraeum*’s bark are utilized to prepare syrup to treat throat inflammation by people of African ancestry in “quilombolas”, small villages reminiscents of African slaves in the Amapa state. Despite the medicinal properties of *Hymenelobium petraeum*, it is well-known the utilization of the wood to build houses and to manufacture furnitures due to its spoil resistance, hardness, and beautiful colour. Seeds of *Vatairea guianensis* found floating in the innumerous rivers crossing the Amapa state, are collected by local people to prepare an unguent to use for dermatological mycosis. The latex extracted from *Symphonia globulifera* is utilized by the Amazonian natives for the treatment of arthritis, despite the wood of this plant be utilized for house construction and furniture manufacture. In spite of all folklore of these plants, many of them are under the risk of extinction because of the economic value represented by the products obtained from these plants. Therefore, the domestication and rational utilization of these plants are urgent and demand a considerable effort of scientists and government.
